# Patient Delay in Initiating Tuberculosis Treatment and Associated Factors in Oromia Special Zone, Amhara Region

**DOI:** 10.1155/2020/6726798

**Published:** 2020-06-11

**Authors:** Muhammed Abdu, Awraris Balchut, Eshetu Girma, Wondwosen Mebratu

**Affiliations:** ^1^Wollo University, Ethiopia; ^2^Debre Birhan University, Ethiopia

## Abstract

**Background:**

Tuberculosis (TB) is a major global public health problem. The disease is a leading cause of morbidity and mortality in Ethiopia. Early identification of cases and commencement of effective chemotherapy is an effective method to control the spread of tuberculosis. Delay in diagnosis and starting tuberculosis treatment increases severity, risk of mortality, and transmission of the disease in the community.

**Objective:**

The purpose of this study is to assess the magnitude of patient delay in initiating tuberculosis treatment and its associated factors among tuberculosis patients in health facilities of Oromia Special Zone, Ethiopia.

**Methods:**

A facility-based cross-sectional study was conducted in Oromia Special Zone. Data were collected using pretested questionnaires from patients with tuberculosis who are on treatment during the study period. The simple random sampling method was used to select health facilities and study participants. Data were entered using Epi Info version 7.2 and analyzed by SPSS version 23. Bivariate and multivariate logistic regression analyses were used to see the significance of association between the outcome and independent variables. A *P* value < 0.05 was considered statistically significant.

**Results:**

Three hundred and eighty-seven tuberculosis patients aged 18 years and above enrolled in the study. Among these, 223 (57.6%) were males, 194 (50.1%) were married, and 206 (53.2%) lived in rural areas. The mean age of respondents was 35 years. The median patient delay was 35 (IQR = 30) days, and 54.4% of patients seek their first consultation after 21 days. Patients who have a basic schooling level (AOR = 0.45, 95% CI: 0.23, 0.89) compared with the college/university level, long distance greater than 10 km (AOR = 3.23, 95% CI: 1.97, 5.42), seeking treatment from informal source and private drug stores (AOR = 3.01, 95% CI: 1.52, 5.95), extrapulmonary tuberculosis (AOR = 2.30, 95% CI: 1.26, 4.23), and poor knowledge about tuberculosis (AOR = 1.58, 95% CI: 1.01, 2.49) were associated factors that predict patient delay. *Conclusion and Recommendation*. A significant proportion of tuberculosis patients delayed to seek treatment. Health promotion and education involving different stake holders will make the community create awareness about tuberculosis that could help reduce delays in initiating tuberculosis treatment.

## 1. Introduction

### 1.1. Background

Tuberculosis (TB) is an infectious disease caused by Mycobacterium tuberculosis, a rod-shaped bacillus called “acid fast”; occasionally, the disease can also be caused by Mycobacterium bovis and Mycobacterium africanum [1]. TB is a major public health problem throughout the world, and also, a third of the world's population is estimated to be infected with tubercle bacilli, hence at risk of developing active disease [2].

According to the 2016 WHO report, in 2015, there were an estimated 10.4 million new TB cases of which 5.9 million were children [3]. The advances in TB prevention and control program lead to a decrease in mortality according to the Millennium Development Goals (MDG). In all, effective diagnosis and treatment of TB saved an estimated 43 million infectiousness, drug resistance, relapse, and even death due to TB [4, 5].

The related literature has revealed that patients' first presentation to health facilities is associated with geographical condition, health service, being unemployed, distance, age, sex, poverty, educational status, rural resident, awareness of TB, self-treatment, and stigma [4–6]. Delay in the diagnosis and start of effective treatment of TB patients result in a prolonged period of infectivity in the community and health care workers. On average, an untreated smear-positive TB patient can infect 10 contacts annually and over 20 during the natural history of the disease until death [6–8].

### 1.2. Statement of the Problem

The End TB Strategy targets to ensure that no family is burdened with catastrophic expenses due to TB [2]. The Sustainable Development Goals (SDGs) for 2030 adopted by the United Nations in 2015 targets to end the global TB epidemic. The WHO End TB Strategy, approved by the World Health Assembly in 2014, calls for a 90% reduction in TB deaths and an 80% reduction in the TB incidence rate by 2030, compared with 2015 [3].

Ethiopia is among the 30 High TB, HIV, and MDR-TB Burden Countries that accounted for 80% of all estimated TB cases worldwide, with an annual estimated TB incidence of 207/100,000 populations and death rate of 33 per 100,000 populations for 2014 [2]. The DR-TB sentinel report of Ethiopia in 2013 showed that the prevalence of MDR-TB is 2.3% among new TB cases and 17.8% among previously treated TB cases [2].

Studies on different parts of the world showed that the magnitude or duration of patient delay was 53.2% in Nepal [4], 72% with greater than 30 days in Colombia [5], 34.8% with a median delay of 32 days in Turkey [7], 65.5% in Iran [9], and 61 and 30 days in Peruvian Amazon and Thailand [10, 11], respectively. In the African region, patient delay, the time from onset of symptom to first consultation to modern health care, is alarmingly prolonged with studies reporting from 71% in Tanzania [12], 61% in Nigeria [13], and 48% in Zimbabwe [14], and also, some studies in Ethiopia showed that there was a patient delay of 62.3% in the North Wollo zone [15], 62% and 52.4% in Bahir Dar town, respectively [16, 17], and 42% in Addis Ababa [18].

Early diagnosis and prompt effective therapy form the key elements of the tuberculosis control program. Delay in diagnosis results in increased infectivity in the community, and it is estimated that an untreated smear-positive patient can infect, on average, 10 contacts annually and over 20 during the natural history of the disease until death [6]. Delay in tuberculosis diagnosis may also lead to a more advanced disease state at presentation, which contributes to late sequelae and overall mortality. Smear-positive cases are more likely to infect other individuals [6].

According to the MOH of Ethiopia hospital statistics data, tuberculosis is the leading cause of morbidity, the third cause of hospital admission (after deliveries and malaria), and the second cause of death in Ethiopia, after malaria [19]. The key elements of TB control and prevention programs are early diagnosis and prompt initiation of effective chemotherapy. Therefore, any delay in diagnosis and consequently treatment of TB patients not only increases infectivity in the community but also may lead to a more advance disease state which may result in more complication and expose them to a higher risk of death [6].

Thus, there are many factors that need to be addressed in order to achieve the goal of reducing the TB burden. As far as the researcher knowledge, these types of studies have not been conducted in the study area. Therefore, this study is aimed at assessing the magnitude of delay for the first consultation and associated factors among tuberculosis patients in Oromia Special Zone.

## 2. Literature Review

Early tuberculosis treatment is aimed at curing the TB patient and restoring quality of life and productivity, preventing death from active TB or its late effects, preventing relapse of TB, preventing the development and transmission of drug resistance, and decreasing TB transmission to others [1].

The End TB Strategy has three high-level indicators; these are the TB incidence rate, the absolute number of TB deaths, and the percentage of TB patients and their households that experience catastrophic costs as a result of TB disease. Targets for these indicators have been set for 2030 and 2035, with accompanying milestones for 2020 and 2025 [3].

In 1993, the World Health Organization (WHO) declared a state of global emergency for tuberculosis (TB), due to the steady increase of the disease worldwide. In 1995, the DOTs (directly observed treatment, short course) strategy was established as the key intervention to achieve tuberculosis control worldwide [6]. Delay diagnosis or incomplete treatment results in prolonged infectiousness, drug resistance, relapse, and even death. Patients with undiagnosed pulmonary TB primarily act as reservoirs for transmission. The contagion parameter suggests that, where TB is endemic, each infectious case will result in 20–28 secondary infections [4].

In order to remove the threat of TB, communities need to be empowered through awareness of primary issues and healthy behaviors. The TB epidemic cannot be addressed without involving those most affected by the disease, and the resulting consequences of their sickness. The communities can help provide practical solutions to the problems many people face when they fall ill and need diagnosis and proper care as well as lead to more interventions by health care professionals [20].

### 2.1. Magnitude of Patient Delay

An analytical cross-sectional study conducted in Nepal showed that the magnitude of patient delay was 53.2% with a median delay of 32 days [4]. The study conducted in Colombia, in 1456 new smear-positive pulmonary tuberculosis patients, shows that 72% of the patient had a delay > 30 days between symptom onset and treatment start and 28% had a 3+ bacillary load at diagnosis [5].

A study done in Iran revealed that 65.5% of cases had received delayed treatment with the mean time between onset of symptom, diagnosis, and treatment of 73 days [9]. A cross-sectional quantitative survey done in Peruvian Amazon showed that the median delay from symptom onset to seeking diagnostic testing was 61 days [10]. Only 31.2% of the study subjects took longer than 30 days to seek a medical consultation in Nonthaburi, Thailand [11]. Another study done in China revealed that 95.5% of patients self-presented for evaluation of the illness after a median 58 days of delay after symptom began [21]. Similar studies done in Pradesh India and Hong Kong show that the median patient delays were 15 and 20 days [22, 23].

A community-based study conducted in India indicates that the mean patient delay was 29 days [22]. Another study conducted in Ibadan, Nigeria, showed that 61.8% of patients delay for more than a median of 30 days [13]. The study conducted in Zimbabwe showed that 48% of patients had a delay with a median of 28 days [14]. Similar studies done in Sudan and Ghana revealed that the median patient delay was 27.2 and 59 days, respectively [8, 24].

The studies conducted in Jimma zone, southwest Ethiopia, and East Wollega, western Ethiopia, showed that the median durations of patient delay for treatment were 32 and 28 days [25, 26]. Another study done in Bahir Dar town and pastoralist communities in Bale zone, Southeast Ethiopia, revealed that the median patient delay was 30 and 63 days [17, 27]. An institution-based cross-sectional study done in North Wollo zone of Amhara region revealed that the median patient delay was 36 days and 62.3% when the patient seek their first consultation after 30 days of cut-off point [15].

### 2.2. Factors That Affect Patient Delay

Residence in the rural areas, DOTs center located more than 5 km away from their residence, and unemployed patients were associated factors with unacceptable patient delay in Nepal [4]. The study conducted in Colombia showed that being uninsured, having unknown HIV status, and coming from a neighborhood with NTP-employed community health workers were significantly associated with patient delay [5], while a similar study conducted in Iran indicates that female gender, smoking, and receiving immunosuppressive drugs were associated with a longer delay time [9].

Similar studies in Thailand and India showed that living in urban area and low family income were associated with patient delay, respectively [11, 22]. A qualitative study done in Peru revealed that the reasons for treatment seeking delay were lack of knowledge and confusion of TB symptoms, fear and embarrassment of receiving TB diagnosis, and patient tendency to self-medicate prior to seeking formal medical attention [28]. Another study done in India revealed that male sex, large family size, completed secondary school, and good knowledge were associated with patient delay [29].

A similar study done in Ibadan, Nigeria, indicates that female sex, place of residence, age group > 45 as compared to ≤45 years, and reported stigma were significantly associated with longer patient delay [13]. Delay in Cameroon is significantly associated with being the main income earner, the belief that TB is stigmatizing, and the use of traditional medicine [30]. The study conducted in Nigeria revealed that patient medicine dealer, private hospital, prayer house, and traditional healer were associated with patient delay [13].

The study conducted in North Wollo zone of Amhara region showed that long distance, rural residence, seeking treatment from traditional healers, and poor knowledge about TB were associated factors that predict patient delay [15]. Another study done in Bahir Dar town showed that illiteracy, having extrapulmonary TB, living greater than a distance of 10 km from TB service in health facilities, and prior visit to holy water, with traditional healer, and with private drug store/pharmacy were determinants of patient delay for the first consultation in health facilities [17]. Another study done in Addis Ababa revealed that patient delay was associated with distance and presence of TB-associated stigma [18]. Tuberculosis (TB) patient delay in Jimma zone is associated with being divorced and uneducated [25]. A similar study done in Wollega showed that patients from urban areas were 46% more likely to present to health care providers and female patients were more delayed to present to health providers than their male counterparts [26]. The study conducted in Mekelle revealed that smear-positive pulmonary TB disease, rural residence, illiteracy, lack of awareness/misperception of cause of pulmonary TB, prior treatment with holy water, treatment by private practitioner, and treatment by drug vendors were associated with patient delay [31].

### 2.3. Significance of the Study

A good understanding of the predictors of patient delay is important in the TB control program. There are so many strategies performed in Ethiopia like TB screening activities by health extension workers, contact screening, and screening at health facilities [2]. Despite efforts made through different methods to sensitize the community with warning symptoms of TB, still, there is a significant delay in health care seeking among TB patients. Information from this study will be useful for policy makers and health departments at different levels in developing interventions aimed at improving early identification of TB cases so as to initiate effective chemotherapy and check disease transmission in the population. It is also useful to others who do studies in this area.

## 3. Objectives of the Study

The objectives of this study were to determine the magnitude of patient delay in initiating TB treatment and identify factors associated with patient delay in initiating TB treatment in Oromia Special Zone.

## 4. Methods

### 4.1. Study Design

A facility based cross-sectional study was conducted.

### 4.2. Study Area and Study Period

The study was conducted in Oromia Special Zone, which is one of the eleven zones of the Amhara region. The zone has a population of 558060 in 2018 and five woredas and two town administrations. In the zone, 27 health centers provide TB treatment service, and one hospital provides smear microscopy, radiographic, and GeneXpert service.

### 4.3. Source Population

#### 4.3.1. Source Population

All TB patients were enrolled one year prior to the study period and on treatment under DOTs program, that is, bacteriologically confirmed (with culture, GeneXpert, and smear), clinically diagnosed, and extrapulmonary patients.

#### 4.3.2. Inclusion and Exclusion Criteria

*(1) Inclusion Criteria*. All tuberculosis patients enrolled and on treatment during the period of data collection were included.

*(2) Exclusion Criteria*. Patients who are critically ill, have treatment failure and relapse, lost to follow-up, unable to respond, and aged <18 years were excluded.

### 4.4. Sample Size and Sampling Procedure

#### 4.4.1. Ssample Size Determination

The sample size was determined using the formula for estimation of single population proportion where *Z*_*α*/2_ at 95% CI (1.96), *d* marginal error 0.05, and *P* proportional obtained from the previous study done in Bahir Dar (0.52) [17]:

(1) $$\begin{array}{c} n=\frac{{\left(Z_{\alpha /2}\right)}^{2}*P\left(1-p\right)}{d^{2}},\\ n=\frac{{\left(1.96\right)}^{2}\left(0.52\right)\left(0.48\right)}{{\left(0.05\right)}^{2}}=384. \end{array}$$

With 5% nonresponse rate, the total sample size of the study was 403, by using the second objective, calculated by Epi Info statistical software (Table 1).

Therefore, the final sample size of the study was 403 which was computed based on the assumption of the single population proportion formula since this provided the larger sample size.

#### 4.4.2. Sampling Technique

Among a total of 28 health facilities in the zone that provides diagnostic and treatment services for DOTs, 22 health facilities were selected randomly. Proportional allocation of patients was made according to the number of TB patients in the selected health facility. Then, all TB patients in the selected health facilities during the study period were included in the study consecutively until the intended sample size was achieved.

### 4.5. Study Variable

#### 4.5.1. Dependent Variable

The dependent variable is delay in seeking care for tuberculosis patients.

#### 4.5.2. Independent Variables

The independent variables were sociodemographic (age, sex, residence, occupation, religion, etc.) variables, health-seeking behavior (treatment sought before the first visit at public HFs, distance to access HFs), health status (type of TB disease (BC/PTB, CD/PTB, and EP) and HIV status (positive, negative)), knowledge of TB disease (the type of disease, its causes, curability, and type of antituberculosis drugs and duration of treatment), and stigma related to TB disease (feeling ashamed of having tuberculosis, having to hide tuberculosis diagnosis from others, delay to seek treatment due to fear of being diagnosed with TB, isolation due to tuberculosis, tuberculosis affecting the relationship with others, and fear of TB/HIV coinfection).

### 4.6. Operational Definitions

1. *Patient Delay*. It is the time interval from the first onset of at least one or more symptom to the first visit to health facility, and a time longer than 21 days was considered indicative of patient delay [16, 18, 27].
2. *The Onset of Symptom*. It is the day when the patient first becomes aware of the symptom or symptoms.
3. *Knowledge*. Variables measuring knowledge were asked from the patient. These included knowledge about the type of disease, its causes, curability, and type of antituberculosis drugs and duration of treatment. Those individuals who score above the mean level were considered having good knowledge.
4. *Stigma*. Variables measuring stigma were assessed using the Likert scale of 10 questions (1 strongly disagree, 2 disagree, 3 agree, and 4 strongly agree).

### 4.7. Data Collection Techniques

#### 4.7.1. Data Collection Instruments and Procedure

An interview schedule was used to capture data from tuberculosis patients attending TB clinics. Some of the statements used were adapted from the “Diagnostic and treatment delay in tuberculosis, in seven countries of the Eastern Mediterranean Region” used by WHO [6]. Consecutive patients were enrolled after leaving the consultation room. They were asked for consent to be interviewed. Before conducting the data collection process, data collectors, 17 diploma nurse and 5 BSc health workers from the health facility currently working in DOTs clinic (TB clinics) and three supervisors who are TB officers, were recruited.

### 4.8. Data Quality Management

The questionnaire was first prepared in English and translated to the local language, Afan Oromo and Amharic, and again back translated back to English to ensure the consistency of the questions. The data collectors and supervisors were trained for one day on how to collect data by using structured questionnaires and general information about the contents of the questionnaires and consent forms. Data were collected with close supervision by a principal investigator and supervisors. The questionnaire was pretested on 5% of the sample size in Oromia Special Zone in similar health facilities, which were not included in the sample, and necessary modifications were made whenever needed.

### 4.9. Data Processing Analysis Procedure

Completed quantitative data were entered into Epi Info version 7.2 software. Then, data were exported to SPSS version 23 and checked for inconsistencies and missing values by running frequencies and other data explorations. Inconsistencies and missing values were cleaned by checking the original questionnaire. Description statistics such as mean, median, standard deviation, and percent were used to describe descriptive variables. Binary logistic regression was employed to investigate the association between independent variables and the outcome variable. Variables that show significant association with the delay in the binary logistic regression at *P* value ≤ 0.25 were included in the multivariable logistic regression models to identify independent predictors to patient delay. In the multiple logistic regression models, the model association was considered at *P* value ≤ 0.05.

### 4.10. Ethical Consideration

Ethical approval was obtained from Wollo University College of Medicine and Health Sciences. A support letter was first requested from the Oromia special zonal health department to woreda health offices and support letter from woreda health offices to health facilities. Verbal consent from respondents was obtained before conducting the face-to-face interview. In order to maintain privacy and avoid the fear of stigmatization, health professionals working in TB units who already knew the serostatus of the clients were assigned to be data collectors. Patients were assured that the interview is private and confidential, their participation is voluntary, and their names were not included in the questionnaire. Data collectors were provided proper advice for the respondents regarding any malpractice they have come across after the interview is completed.

## 5. Results

### 5.1. Sociodemographic Characteristics

Three hundred and eighty-seven TB patients with a response rate of 96% were enrolled from 22 treatment and diagnostic centers, whereas the remaining was excluded due to data incompleteness during data analysis. The mean age was 35, years and 226 (58.4%) participants were 18-34 years old and 161 (41.6%) were 35 years and above. In the majority of participants, 57.6% were males, 53.2% lived in rural areas, 81.9% were Muslims, and 50.1% were married and 52.5% did not have any schooling level. Two-thirds of the study participants lived within the 10 km radius to health facilities. The main occupation of the participants was farmer (33.1%), and 13.2% were government or private employees (Table 2).

### 5.2. Clinical Characteristics of TB Patients

Among the respondents, 136 (35.1%) were bacteriologically confirmed PTB, 145 (37.5%) were clinically diagnosed PTB, and 106 (27.4%) were extrapulmonary TB. The majority of the participants (74.7%) had cough/cough with blood as a symptom of tuberculosis, 63% had night sweating, and 58.1% had loss of appetite. Most of them reported multiple symptoms of tuberculosis in this study (Table 3).

### 5.3. Health-Seeking Behaviors of TB Patients

A total of 275 patients (71.1%) had not sought informal care for their symptoms between the onset of their illness and their first consultation at a health facility, while 102 (26.4%) had sought care from at least one informal provider. Twenty-one (5.4%) had been treated with holy/hot water, 28 (7.2%) by traditional healers, and 63 (16.3%) by private drug stores/pharmacies.

The majority of patients (192 or 46.9%) made their first consultation at a health center, 119 (30.7%) attended the nearby clinics, and 78 (19.6%) at a hospital. Most patients (57.6%) decided to visit health facilities by themselves; forty-two percent of the patients visited a health facility because they were advised by members of their family or health workers. In this study, 43.4% of patients had chat chewing habit, 10.1% had smoking habit, and 2.3% reported alcohol consumption before tuberculosis diagnosis (Table 4).

### 5.4. Patient Delay

Overall, 211 (54.5%) with 95% CI (49%, 62%) of patients did seek medical advice after 21 days of the onset of their illness. The median delay from onset of symptoms to first visit of a health provider was 35 (IQR = 30) days, range (23-360 days). A total of 353 patients (91.2%) came within two months, and 34 patients (8.8%) came after 90 days. Of all delays, 28.9% were BCPTB, 33.6% were CDPTB, and 36% were EPTB patients. Patient delay among women was 43.1% and men 56.9%. Based on the place of residence, 113 (58.3%) of urban and 71.5% of rural residents were delayed. Among the respondents, 54.9% with poor knowledge was delayed.

On the bivariate analysis, educational status, basic schooling level, and distance to health facility had significantly increased the risk of delay in seeking health care of TB treatment. Respondents with poor knowledge about tuberculosis and those with prior attendance at holy/hot water and nearby drug stores were associated with patient delay.

Having extrapulmonary TB was 2 times more likely to cause delay in seeking health care of TB treatment. No significant association was found between prolonged patient delay with sex, age, marital status, HIV status, and occupation.

On multivariant analysis, respondents with basic schooling level (AOR = 0.45, 95% CI: 0.23, 0.89) and patients who lived beyond a 10-kilometer radius from health facilities (AOR = 3.23, 95% CI: 1.97, 5.42) were three times more delayed than those within the 10 km radius. The use of prior treatment with holy/hot water (AOR = 3.62, 95% CI: 1.11, 11.84) and from private drug store/pharmacy (AOR = 3.01, 95% CI: 1.52, 5.95) was also significantly associated with patient delay.

Extrapulmonary TB patients were two times more likely to be delayed than those study participants with bacteriologically confirmed pulmonary TB (AOR = 2.30, 95% CI: 1.26, 4.23). Having poor knowledge about tuberculosis was another important factor found to cause patient delay in seeking health care for TB treatment (AOR = 1.58, 95% CI: 1.01, 2.49) (Table 5).

## 6. Discussions

Early detection of cases and treating TB patients are one of the strategies of WHO to reduce the disease morbidity and mortality throughout the world [3]. In this study, it was observed that a significant proportion of patients delayed more than the acceptable level for patient delay. The magnitude of patient delay in seeking health care was 54.5%, and this is almost similar with studies conducted in Nepal 53.2% [4], Bahir Dar 52.4% [17], and Tigray 53% [31], and it is greater than the previous studies in Thailand 31.2% [11] and Addis Ababa 42.1% [18]. But the result of this study is lower than that of the study done in pastoralist communities in Bale zone 96% [27]. The possible reason for this difference may be due to the study setting and topographic conditions.

The overall median patient delay in this study was 35 days with a range of 23-360 days, which was consistent with a study done in North Wollo zone 36 days [15]. But it was somewhat longer than a study done in northwest Ethiopia, Amhara region, with a median of 30 days Bahir Dar town [16, 17] and east Wollega, eastern Ethiopia [25]. It was much longer than previous studies in Pradesh, India, [32] with a median delay of 15 days and Hong Kong [23] with a median delay of 20 days. But it was much shorter than the previous study in Bale zone a median delay of 63 days [27]. The possible suggestion for differences in delay could be probably by cultural, low socioeconomic status, and lack of information about the availability of free tuberculosis treatment.

Long walking distance between health facility and patient home was a risk factor for seeking health care of tuberculosis treatment. Respondents, who live beyond 10 kilometers from health facilities, have 23% times higher delay in seeking health care as compared to those who live within 10 kilometers. This result was in line with studies done in Nepal [4], North Wollo [15], Bahir Dar [17], and Addis Ababa [18]. This might be low inaccessibly in health service and scattered settlement of population.

Regarding the respondent educational level, having basic schooling level was 45% less likely to delay compared to those who have college or university level. This result is different with studies done in Peruvian Amazon [10], Tanzania [33], Bahir Dar [17], and Bale zone [27]. This difference might be the availability of community health insurance made respondents, who have basic schooling level, seek health care in health facilities at the soonest possible time.

The use of an informal treatment provider was also another strong predictor of patient delay in this study. This is in line with previous studies in Peru [28], Nigeria [34], Bahir Dar town [17], and Tigray [31]. Patients who used holy/hot water as a first consultation were four times more likely to delay in seeking health care for tuberculosis treatment compared to those who did not use. This is consistent with previous studies in North Wollo [15] and Bahir Dar [17]. This might be low reliance of treatment in health facilities and high cultural beliefs of respondents on treatment of TB.

Respondents, whose first consultation in private drug stores or pharmacies, were three times more likely to delay in seeking health care of treatment compared to those who did not. This is in line with the study conducted in Nigeria [34], North Wollo zone [15], and Bahir Dar town [17]. The probable reason for this could be that private drug stores or pharmacies have an advantage of availability in most areas and also, they do not charge for cards and other consultation services.

Extrapulmonary TB constituted 27.4% of all cases in this study. Respondents, with extrapulmonary TB, were two times more likely to delay as compared to bacteriologically confirmed pulmonary TB patients. This study was consistent with the studies in Bahir Dar town [17]. However, findings from studies in European countries reveal that extrapulmonary TB is a more rare form of TB and it is more frequent in African patients [35, 36]. This might be due to a variety of symptoms, could easily be over looked, gradual progress, and less sever clinical manifestation at the beginning. Another probable reason might be that patients with extrapulmonary TB feel reassured about their health conditions and do not proceed with necessary investigation to diagnose in peripheral health facilities.

Having good knowledge about tuberculosis is a very important issue for early seeking of medical consultation. In this study, 49.4% of respondents have poor knowledge about tuberculosis. Respondents, with poor knowledge about TB, were 58% more likely to delay in seeking health care of tuberculosis treatment as compared to those who had good knowledge. This is in line with previous studies conducted in North Wollo zone [15], Amhara region, and WHO in east Mediterranean study in Syria Arab republic [6]. This difference might be dependent upon the respondent's knowledge regarding the TB disease for early seeking of medical care.

Patient delay was not found to be significantly associated with age and sex in this study. Similar findings were reported in North Wollo [15] and Addis Ababa [18]. However, studies conducted in Iran [35]; Ibadan, Nigeria [13]; and Wollega [26] reported that females tend to seek health care lately compared to males. This might be due to work overload at home and the limited role of women's decision making power. Similarly, older age was found to be significantly associated with patient delay in India [32] and Ibadan, Nigeria [13]. This difference might be due to older age people are physically and economically dependent with others in their life.

In this study, stigma was not associated with patient delay in seeking care for TB treatment. This is similar with studies conducted in Bahir Dar [17] and Wollega, eastern Ethiopia [26]. Other several studies showed stigma attached to the TB patient is a contextual factor associated to delay seeking of the health services in Cameroon [30], Ghana [24], which is not the case in this study.

In Ethiopia, all patients with a diagnosis of TB are recommended to be tested for HIV [2]. In this study, 98.9% of respondents were checked for HIV. In this study, those with HIV coinfection had a slightly longer delay in seeking treatment but this was not significant; this is similar to studies in Thailand [5], Tanzania [33], and Nigeria [34]. This may be due to shortage of financial resources in the course of seeking treatment for their HIV care.

The strength of this study is it includes 70% of the health facilities that provide DOTs services in the study area and also participate both public and private health facilities. However, there was some degree of recall bias while patients were trying to remember the symptoms to approximate days of the beginning of the symptoms and starting TB treatment. To minimize this limitation, local religious calendar methods and public holidays, national celebrations, and agricultural events were used to facilitate patient recall to collect information.

The result of this study revealed that majority of TB patients in this study area did not present within 21 days from onset of illness to a health facility. Educational status having basic schooling level, distance greater than 10 kilometers, first point of contact at holy/hot water, private drug stores or pharmacies, extrapulmonary TB, and poor knowledge about tuberculosis were found to be predictors for prolonged patient delay for first consultation in health facilities. Patient delays were found to be longer, suggesting the need for increased advocacy, communication, and social mobilization efforts targeted at informing the general population to seek health services early when they experience symptoms suggestive of TB. Health education sessions that emphasized the cause, the availability of treatment, and the curability of tuberculosis should be designed and provided to raise awareness.

Based on results found in this study, the authors recommended that the community should be sensitized continuously on seeking appropriate health care and sensitization programs by using socially and culturally convenient media of communication to ensure that the whole community is reached. The health department of the local government should orient religious leaders, private drug stores or pharmacies, and traditional healers to refer symptomatic people for tuberculosis screening and treatment. A community-based study can be done to capture symptomatic individuals who are not attending health facilities.

## Figures and Tables

**Table 1:** Sample size calculation by TB patient delay-associated factors.

Associated factors	Prevalence	Power	Confidence interval	AOR	Sample size	Including 5%	Reference
TB-associated stigma	42.1	80	95	2.1	262	275	[18]
Informal treatment source (holy water)	62.3	80	95	2.58	228	239	[15]
Educational level (illiterate)	62	80	95	3.73	144	151	[16]
Distance to HFs	52.4	80	95	3.15	114	120	[17]

**Table 2:** Characteristics of tuberculosis patients in DOTs clinics of Oromia zone, Northeast Ethiopia, 2018.

Variables	Frequency (*n* = 387)	Percent
Sex		
Male	223	57.6
Female	164	42.4
Age		
18-34	226	58.4
35-50	98	25.3
≥51	63	16.3
Place of residence		
Rural	206	53.2
Urban	181	46.8
Marital status		
Married	194	50.1
Single	150	38.8
Divorced	23	5.9
Widowed	20	5.2
Educational status		
Did not have any schooling level	203	52.2
Did have basic schooling level	69	17.8
Primary	57	14.8
Secondary	36	9.3
College/university	22	5.7
Religion		
Muslim	317	81.9
Orthodox	47	12.1
Protestant	23	5.9
Ethnic group		
Oromia	249	64.3
Amhara	136	34.9
Tigre	3	0.8

**Table 3:** Clinical characteristics of TB patients in Oromia zone, Northeast Ethiopia, 2018.

Variables	*N* (%)
Types of symptom	
Cough/cough with blood	289 (74.4%)
Night sweating	244 (63.0%)
Loss of appetite	225 (58.1%)
Fever	216 (55.8%)
Chest pain	192 (49.6%)
Weight loss	163 (42.1%)
TB type	
CDPTB	145 (37.5%)
BCPTB	136 (35.1%)
EPTB	106 (27.4%)
HIV status	
Negative	355 (91.7%)
Positive	28 (7.2%)
Unknown	4
Other comorbid conditions	
No	376 (97.2)
Diabetes	6
Hypertension	5
Smoking habit	
No	348 (89.9%)
Yes	39 (10.1)
Chat chewing habit	
No	219 (56.6%)
Yes	168 (43.4%)

**Table 4:** Health-seeking behaviors of TB patients in Oromia zone, Northeast Ethiopia, 2018.

Variables	*N* (%)
Treatment sought prior to first visit in health facilities	
No prior treatment	275 (71.1%)
Private drug store/pharmacy	63 (16.3%)
Traditional healers	28 (7.2%)
Holy/hot water	21 (5.4)
Decision to visit the first health facility was made by	
Patients themselves	223 (57.6%)
Family members	135 (34.9%)
Health workers	29 (7.5%)
Health facility visited at first consultation	
Health centers	192 (49.6%)
Clinics	119 (30.7%)
Hospitals	76 (19.6%)
Knowledge about TB	
Good knowledge	196 (50.6%)
Poor knowledge	191 (49.4%)
TB-associated stigma	
Low stigma	223 (57.6%)
High stigma	164 (42.4%)

**Table 5:** Factors associated with patient delay in DOTs clinics of Oromia Zone, Northeast Ethiopia, 2018.

Variables	Patient delay		Unadjusted and adjusted OR		*P* values
	No delay	Delay	COR (95% CI)	AOR (95% CI)	
Educational status					
College/university	14	8	1	1	
Did not have any schooling level	73	130	3.12 [1.25, 7.77]	0.74 [0.39, 1.39]	
Have basic schooling level	35	34	1.7 [0.63, 4.57]	0.45 [0.23, 0.89]	0.02^∗^
Primary	37	20	0.95 [0.34, 2.64]	0.67 [0.29, 1.56]	
Secondary	17	19	1.96 [0.65, 5.80]	0.49 [0.17, 1.41]	
Place of residence					
Urban	93	88	1	1	
Rural	83	123	1.57 [1.05, 2.34]	0.99 [0.57, 1.56]	
Distance to the nearest health facilities					
<10 km	142	113	1	1	
≥10 km	34	98	3.62 [2.28, 5.75]	3.23 [1.97, 5.42]	0.001^∗∗∗^
Treatment sought prior to first visit in health facilities					
No prior treatment	149	126	1	1	
Holy/hot water	4	7	5.03 [1.65, 15.32]	3.62 [1.11, 11.84]	0.034^∗^
Private drug store/pharmacy	15	48	3.78 [2.02, 7.08]	3.01 [1.52, 5.95]	0.001^∗∗∗^
Traditional healers	8	20	2.96 [1.26, 6.94]	2.14 [0.81, 5.60]	
TB type					
BCPTB	75	61	1	1	
CDPTB	74	71	1.18 [0.74, 1.89]	1.19 [0.71, 1.99]	
EPTB	27	79	3.59 [2.07, 6.25]	2.30 [1.26, 4.23]	0.001^∗∗∗^
Knowledge about TB					
Good knowledge	105	95	1	1	
Poor knowledge	71	116	1.81 [1.20, 2.71]	1.58 [1.01, 2.49]	0.04^∗^

AOR = adjusted odds ratio; COR = crude odds ratio; CI = confidence interval. N.B. ^∗^*P* value ≤ 0.05, ^∗∗∗^*P* value ≤ 0.001. Coding of dependent variable: delay = 1 and no delayed = 0.

## Data Availability

The data will be available on request.
