# Corrigendum to “Patient Delay in Initiating Tuberculosis Treatment and Associated Factors in Oromia Special Zone, Amhara Region”

**DOI:** 10.1155/2020/9505083

**Published:** 2020-11-10

**Authors:** Muhammed Abdu, Awraris Balchut, Eshetu Girma, Wondwosen Mebratu

**Affiliations:** ^1^Wollo University, Ethiopia; ^2^Debre Birhan University, Ethiopia; ^3^Department of Preventive Medicine, School of Public Health, Addis Ababa University, Ethiopia

In the article titled “Patient Delay in Initiating Tuberculosis Treatment and Associated Factors in Oromia Special Zone, Amhara Region” [1], an affiliation was omitted in error. This affiliation has been added to the affiliation list above as affiliation 3, and the author affiliations have been corrected.

## References

[1] Muhammed A., Awraris B., Eshetu G., Wondwosen M. (2020). Patient Delay in Initiating Tuberculosis Treatment and Associated Factors in Oromia Special Zone, Amhara Region. *Pulmonary Medicine*.

